# Nonrandom spatial distribution of Neotropic Cormorants (*Phalacrocorax brasilianus*) along a coastal highway in Lima, Peru

**DOI:** 10.1371/journal.pone.0242835

**Published:** 2021-03-01

**Authors:** Sebastián Lozano-Sanllehi, Carlos B. Zavalaga

**Affiliations:** 1 Unidad de Investigación de Ecosistemas Marinos—Grupo Aves Marinas, Universidad Científica del Sur, Lima, Perú; 2 Facultad de Ciencias Ambientales, Universidad Científica del Sur, Lima, Perú; MARE – Marine and Environmental Sciences Centre, PORTUGAL

## Abstract

Neotropic Cormorants (*Phalacrocorax brasilianus*) are common seabirds along the Peruvian coast. They frequently perch on trees, poles and port structures in urban areas, producing guano that builds up in areas of high levels of human activity. Hundreds of Neotropic Cormorants rest on lighting poles and telephone cables along a 12.7 km highway in the coastal strip of the city of Lima, Peru. We hypothesized that the distribution of the cormorants along this highway is clustered and could be associated with physical features of both the coast and the adjacent marine area. Fortnightly or monthly surveys were performed from July 2018 to March 2020 in the Circuito de Playas de la Costa Verde highway. At each survey, cormorants were counted per lighting pole and adjacent telephone cables (collectively, “pole-cable”) at four count hours (0600 h, 1000 h, 1400 h and 1800 h). Our results revealed that daily bird numbers varied from 46 to 457 individuals and that only 17% of the total number of pole-cables (N = 651) was occupied once by at least one individual. The number of cormorants also varied between count hours within the same day (higher numbers at 1000 h and 1400 h). Birds were clustered into a maximum of five hotspots along the highway. According to a model selection criterion, higher numbers of cormorants on pole-cables were associated mainly to a closer distance from these structures to the shoreline and to the surf zone, suggesting that Neotropic Cormorants may select such pole-cables as optimal sites for sighting and receiving cues of prey availability. Based on the results, the use of nonlethal deterrents and the relocation of these birds to other perching structures on nearby groynes could be the most suitable management proposal for the problems caused by their feces.

## Introduction

Human-seabird interactions worldwide have caused drastic changes in the number and distribution of various species of seabirds [1], many of which (42%) are considered by conservation organizations as being threatened with extinction [2, 3]. Conversely, many species of seabirds have benefited from the available food in fishing discards [4–6], the presence of coastal landfills [7, 8] and the aquaculture industry [9, 10]. This has caused seabirds to interact more frequently with humans and in some cases to be considered pests [11, 12]. For example, some species of gulls can spread diseases [13–15], transport pollutants [16], damage urban infrastructure [17, 18], and collide with airplanes [19, 20]. The majority of gulls are generalist predators and often occupy urban areas [11, 18, 21], thus resulting in frequent interactions with humans. Other species of seabirds such as cormorants, which are mainly piscivorous [22, 23], also interact with humans. For example, Neotropic Cormorants (*Phalacrocorax brasilianus*, hereinafter “NECOs”), Double‐crested Cormorants (*P*. *auritus*) and Great Cormorants (*P*. *carbo*) obtain food from fish farms [24, 25] and generate conflicts with fisheries [26]. In Peru, the droppings of cormorants, among other species of seabirds, cause deterioration in port facilities [27]. In coastal cities of Chile, the excretions of NECOs perching in trees and public lighting poles cause nuisance to people and damage to infrastructure and vegetation in parks and avenues [28]. A similar problem with this species occurs in Costa Verde, the coastal strip of the Miraflores Bay in the city of Lima, Peru [29], through which the Circuito de Playas de la Costa Verde (CPCV) highway extends.

The NECO is a species of wide distribution in the American continent, from the southern United States to Cape Horn, Chile [22, 30]. It is present in a wide variety of coastal, Amazonian and high Andean ecosystems [31–33], from sea level to altitudes up to 4800 m above sea level [30, 34]. It feeds in shallow waters [35, 36], both in continental and marine water bodies [22, 37], and its diet is composed of a large variety of prey, mainly benthic fish [38–40]. It frequents places with natural perches such as trees or rocks, but it also uses manmade structures (e.g., poles, cables, buoys) where it performs activities of preening, feather drying and daytime rest [41, 42].

The first records of the presence of NECOs occupying tall trees and lighting poles at the top of the cliffs of Costa Verde date from the mid-1950s [43]. However, over the years, the construction of vehicular roads, beaches, rock groynes and other infrastructure has led these birds to use public lighting poles and telephone cables present along the CPCV highway to rest and preen, resulting in the contact of their feces with the road, cars, infrastructure and passers-by [29, 44]. It is speculated that their droppings could cause public health problems due to their possible content of pathogens [45], as happens in other birds [46], or cause corrosion to vehicles and infrastructure, such as occurs with the Double-crested Cormorant (*P*. *auritus*) in the Columbia River (Astoria) Bridge, United States [47, 48]. Based on the experience of other regions [28], these problems could intensify or move to other areas over the years because Costa Verde is subject to a growing implementation of urban projects.

Even though this environmental problem is not novel in Costa Verde, no management action has been taken so far. This lack of management makes the development of studies and establishment of proposals to mitigate the conflict between NECOs and humans relevant. Different conflict-management measures used for NECOs in the cities of Arica and Iquique, Chile, have had variable results; these include the relocation of colonies, the destruction of nests, the culling of individuals, the use of sound and visual stressors, among others [28]. In the province of Chubut, Argentina, the culling of NECOs and the harassment of their colonies are used as methods against presumed impacts of this species in fishing and aquaculture activities [49].

For the Peruvian case, to determine which management proposals are the most appropriate and in which CPCV-highway sections they should be implemented, it is important first to examine the distribution of the NECOs along the highway and thus identify the aggregation hotspots of these birds by district. Additionally, identification of the factors that determine the preference of the NECOs for perching sites is required for the choice of such management proposals. For these reasons, in this study, fortnightly or monthly counts were performed between 2018 and 2020 on poles and telephone cables that these birds used as perches along the CPCV highway. We hypothesized that the distribution of NECOs in the CPCV highway is not random but clustered, and is associated to places that provide better foraging opportunities for these birds. Therefore, the objectives were (1) to evaluate the spatial distribution and temporal variation of the number of NECOs in the CPCV highway, and (2) to examine the influence of physical features of both Costa Verde and the adjacent marine area in their spatial distribution. Based on the results, management proposals for the environmental problem caused by their excreta were posed. This is the first study aimed at solving problems caused by seabirds in urban-coastal areas of Peru.

## Methods

### Study area

**Fig 1:**
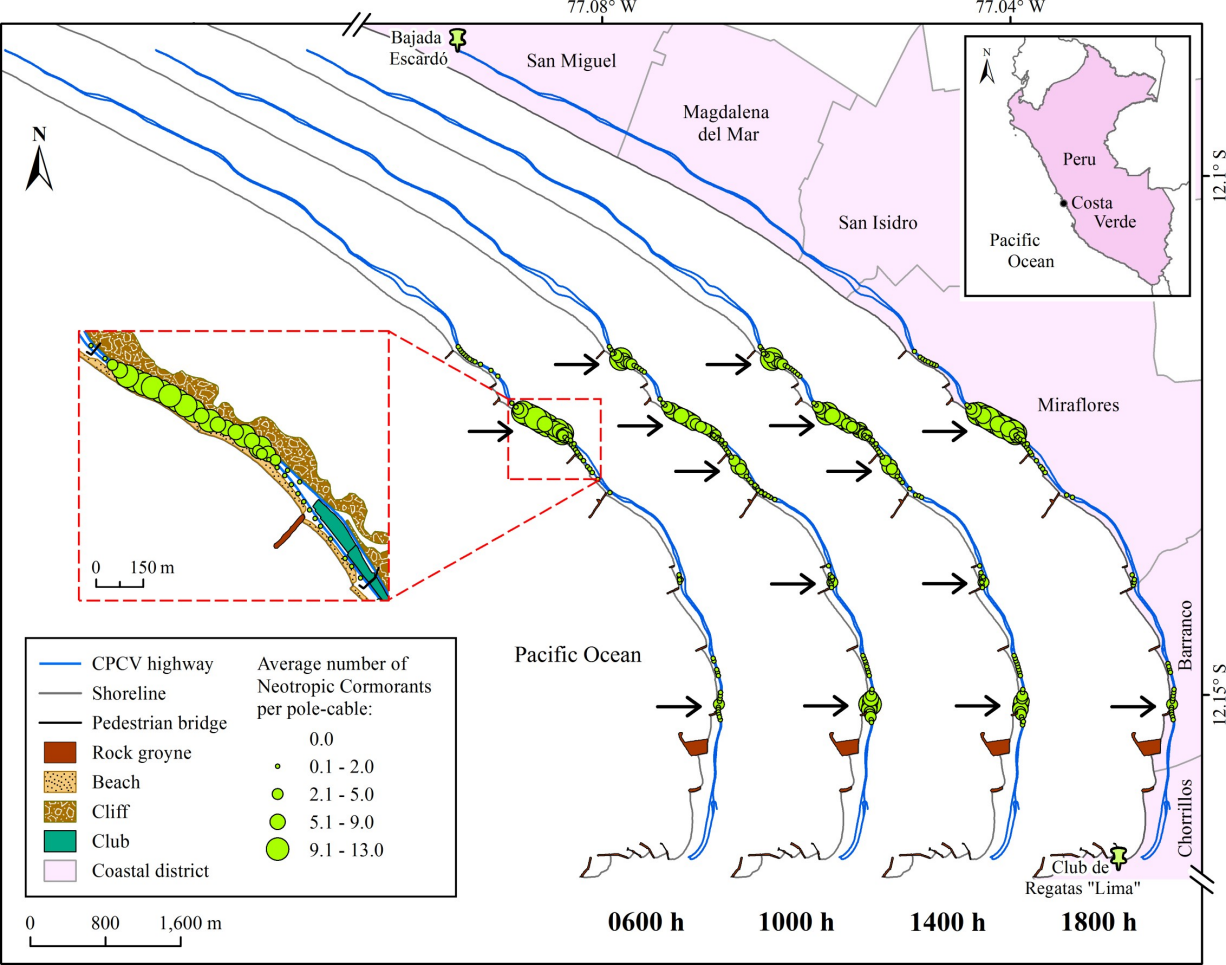
Location of the study area and spatial distribution of the Neotropic Cormorants (*Phalacrocorax brasilianus*) by count hour in the Circuito de Playas de la Costa Verde highway in Lima, Peru. The size of the green circles is a function of the study-period average number of cormorants per pole-cable. The arrows indicate the aggregation hotspots of the birds. The country boundaries layer of the locator map was obtained from [50], with permission from Esri Inc., original copyright 2017.

**Fig 2:**
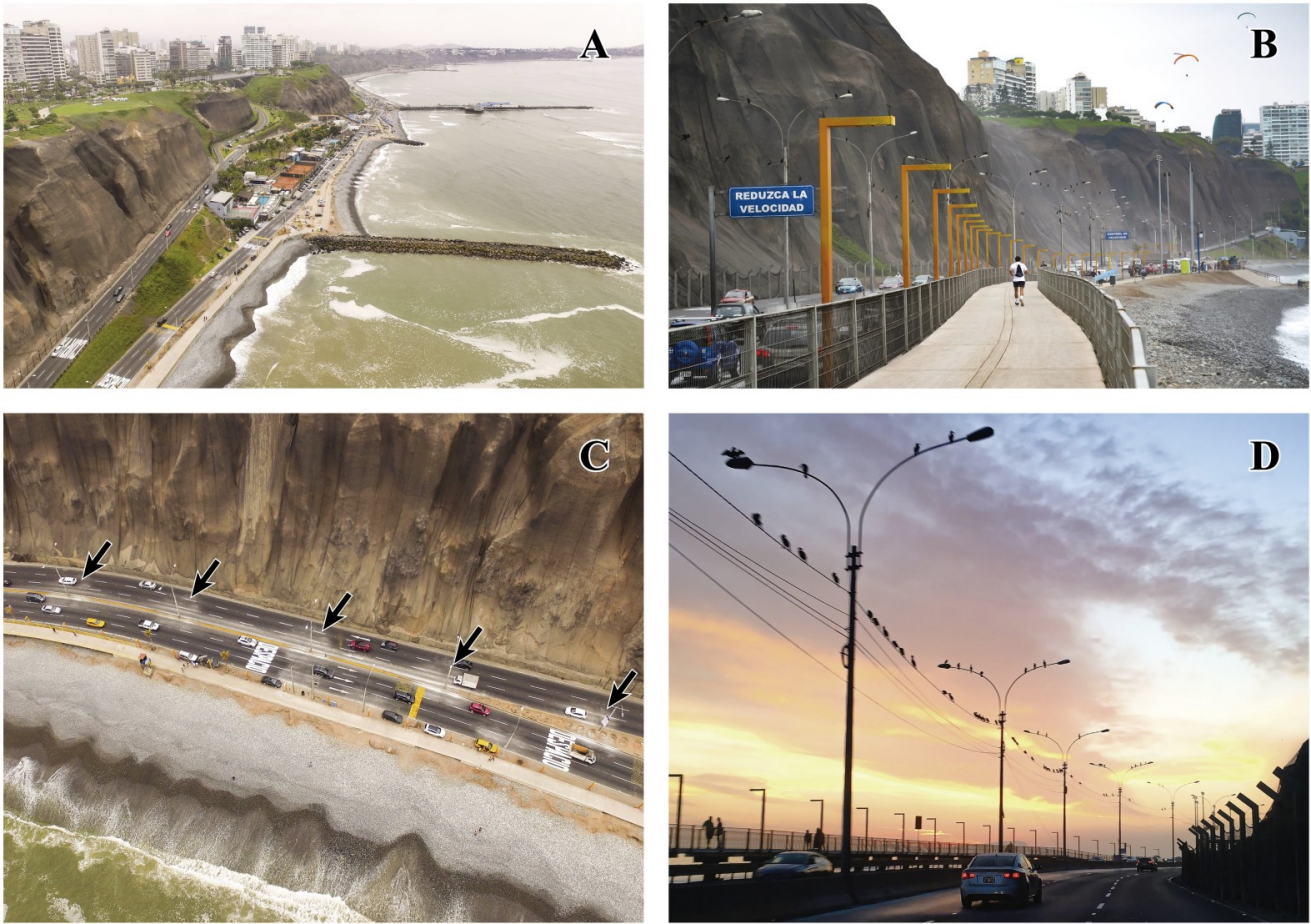
Features of Costa Verde. (A) Cliffs, clubs, groynes and pebble beaches. (B) Infrastructure for pedestrians. (C) Vehicular road with presence of cormorant feces around each lighting pole (pointed by black arrows). (D) Neotropic Cormorants (*Phalacrocorax brasilianus*) perching on lighting poles and telephone cables.

This study was carried out in Costa Verde, the coastal strip of the Miraflores Bay in the city of Lima, Peru (Fig 1). This is a tourist/recreational space with a large influx of vehicles and people throughout the year, especially in summer. It has beaches, parks, sports complexes, restaurants, clubs, parking areas adjacent to the beaches and infrastructure for pedestrian and vehicular traffic (Fig 2A and 2B). The CPCV highway extends along Costa Verde, and is bordered to the east by cliffs up to 100 m high and to the west by sandy, gravel and pebble beaches, usually narrow, with rock groynes perpendicular to the coastline ([44], Fig 2A). This 12.7 km long road includes from the entrance to Club de Regatas “Lima” (club) from the south (12°10’0.2"S, 77°1’48"W) to Bajada Escardó (access road) from the north (12°5’14.4"S, 77°5’38.8"W; Fig 1), and has two to three lanes for vehicular traffic on both the outward and return tracks (Fig 2C). It also features public lighting poles and telephone cables that allow NECOs and other birds, such as gulls and Black Vultures (*Coragyps atratus*, Fig 2D), to perch.

Based on World Imagery images (projected into UTM zone 18S) from the ArcGIS Online service, and using a GARMIN GPSmap 62S® handheld GPS at field, different attributes of Costa Verde were digitized as individual layers using ArcGIS 10.5 software [51]: points (public lighting poles), lines (vehicular roads, shoreline) and polygons (cliffs, groynes, beaches). This allowed the calculation of the position and distance of different attributes required in the data analysis. Each lighting pole was identified with a unique code.

### Counts

Visits to the CPCV highway were made fortnightly (in the middle and at the end of each month), from July 14, 2018 to June 30, 2019, and monthly (at the end of each month), between July 31, 2019 and March 27, 2020. In both cases, four surveys were performed on the day of count: 0600 h, 1000 h, 1400 h and 1800 h, resulting in a total of 96 fortnightly and 33 monthly counts (on March 27, 2020, NECOs were only counted at 1000 h). The 0600 h counts were performed at dawn (0530–0630 h), as soon as the daylight was sufficient to count the NECOs; in contrast, the 1800 h counts were performed as late as possible (1740–1840 h), before the darkness and the glare from the lighting poles would make this task difficult.

The number of NECOs in the CPCV highway was determined by direct count of birds recorded in high-resolution videos (4K/60fps). For this, a GoPro HERO6® camera was attached to the roof of an automobile that moved at a speed <30 km/h. The camera lens pointed to the top of the poles and cables, at a maximum distance of 20 m. At this distance, and with a high-resolution video, there was no count error related to bird-to-bird overlapping. Beaches and other surrounding areas were not included in the counts since they were not occupied by the NECOs during the study period. The counting of individuals was performed per lighting pole and that number was associated with the code of the pole. In the case of the NECOs present on telephone cables, the length of the cable between poles was divided into two equal parts, and the NECOs on each half were assigned to the nearest pole. Therefore, the term “pole-cable” is used hereinafter as a unit of counting and analysis. The Franklin’s Gull (*Leucophaeus pipixcan*), an abundant seasonal migratory species, also present on pole-cables, was also counted.

There were no specific permits and approvals required for data collection since Costa Verde is a public area and no animal experimentation was performed.

### Definition and measurement of explanatory variables

The distribution of lighting poles along the CPCV highway was regular, with a spacing of 30 m from each other. Neither the poles nor the telephone cables had deterrents that prevented birds from occupying them, such as spikes or bird wires. The number of NECOs on pole-cables was examined with regard to variables of physical features of both Costa Verde and the adjacent marine area (<1 km from the shoreline), which could be related to the foraging behavior of this seabird species and the accessibility to its prey. Six variables were measured: Distance from the pole-cables to the shoreline, Distance from the pole-cables to the surf zone, Distance from the pole-cables to the nearest groyne, Perimeter of the nearest groyne, Distance from the shoreline to the 7 m isobath, and Transparency of seawater.

#### Distance from the pole-cables to the shoreline (DS)

The distance from the pole-cables to the shoreline varies along the CPCV highway. Prior to this study, it was observed on several occasions that there was a greater number of NECOs on pole-cables in sections of the highway closer to the shore. Thus, to examine this effect, the minimum distance in meters from each pole-cable to the shoreline (line that passes between the high and low tide marks) was measured in ArcGIS.

#### Distance from the pole-cables to the surf zone (DSZ)

The surf zone is the relatively narrow strip that borders the ocean beaches and extends from the shoreline to the last breaking wave offshore [52]. It is a very dynamic and productive zone, as it holds a wide variety of life forms [53]. In Costa Verde, its width varies along the coast and could represent areas preferred by the NECOs for their foraging activities.

Five satellite images of Costa Verde on dates coinciding with the study period (October and December 2018, March and April 2019, February 2020) were obtained from the historical images of Google Earth Pro 7.3 software [54], and then georeferenced in ArcGIS. On each image, a layer of the shoreline sections in front of a surf zone with a width greater than 70 m (measured from the shoreline to the last breaking wave close to the coast) was digitized. Smaller widths were not taken into account because they were common along the coastline. Once the layers of the five images were generated, they were overlapped. The sections with matches in at least three images were defined as surf zone sections. Finally, the minimum distance from each pole-cable to the nearest surf zone section was measured.

#### Distance from the pole-cables to the nearest groyne (DG)

Coastal protection structures alter the hydrodynamic regimes and depositional processes of the coastal zone [55], which can lead to changes in the habitat, composition and distribution of marine species [56]. Thus, the presence of rock groynes in Costa Verde could generate feeding areas preferred by the NECOs. For the calculation of this variable, the minimum distance from each pole-cable to the base of the nearest groyne was measured in ArcGIS.

#### Perimeter of the nearest groyne (PG)

The hard substrate of groynes allows many marine organisms to settle [56, 57]. Thus, the larger groynes and those whose contour has more contact with the seawater would generate a greater feeding area for the NECOs. For this reason, each pole-cable was assigned the length of the contour (in contact with the seawater) of the nearest groyne.

#### Distance from the shoreline to the 7 m isobath (D7)

According to various authors, NECOs have a preference for areas near the coast [30], where they feed in shallow waters close to the shoreline [35, 49, 58]. In Costa Verde, the number of NECOs on pole-cables could be associated with a preference for shallow marine areas, which are more extensive at a greater distance from the shoreline to the isobaths.

The isobaths of the marine area of Costa Verde were obtained from the digitized nautical chart No. 2237, corresponding to the Miraflores Bay [59]. The minimum distance from the shoreline (in front of each pole-cable) to the 7 m isobath was measured in ArcGIS. The choice of this isobath was based on records of the preference of NECOs to feed in waters with depths less than 7 m on average [36, 60, 61].

#### Transparency of seawater (T)

In general, seabirds tend to congregate in areas of high prey density [62]. However, their access to these depends on variables such as water clarity [63]. Therefore, possible changes in the transparency of seawater could influence the presence of NECOs on pole-cables in the CPCV highway.

This variable was measured between the months of October 2018 and January 2019 (one measurement per month) on dates coinciding with surveys. These measurements were performed with a Secchi disk from a boat in areas with depths between 5 and 8 m and at an approximate minimum distance of 500 m from the shoreline. The presence of waves prevented going further inshore. Data were taken at the beginning, center and end of transects of 400 m perpendicular to the shoreline, in front of five sections with presence and five sections with absence of NECOs (each section of 15 pole-cables). Because both the hotspots of NECOs and the sections of the highway with absence of these birds were highly predictable during the study period, the transects were always situated at the same locations. Finally, the values obtained by transect were averaged, thus obtaining a total of 40 data points of seawater transparency throughout the evaluation period of this variable.

### Analysis of the spatial distribution and temporal variation of the number of NECOs

Count data were examined to determine NECOs’ random or cluster distribution along the CPCV highway and to identify occurrence hotspots. For management purposes, the number of NECOs was analyzed by district (San Miguel, Magdalena del Mar, San Isidro, Miraflores, Barranco and Chorrillos). The district boundaries layer was obtained from the Instituto Geográfico Nacional website [64].

For each count hour (0600 h, 1000 h, 1400 h and 1800 h), the number of NECOs per pole-cable of all counts was averaged across the entire study period. These averages were used in ArcGIS to generate maps and define the aggregation “hotspots” of the NECOs per count hour. A set of consecutive pole-cables with two or more NECOs on each one was considered as a “hotspot”.

To determine whether the spatial distribution of the NECOs was random or clustered, the Moran spatial autocorrelation measure (global Moran’s I) was applied to the averaged data of each count hour using ArcGIS. Likewise, frequency graphs of the number of NECOs per pole-cable and the two-sample Kolmogorov-Smirnov (KS) test (*stats* R package [65]) were used to assess and compare the level of aggregation between count hours.

The temporal variation of the number of NECOs among count hours (0600 h, 1000 h, 1400 h and 1800 h) and among seasons (austral summer, autumn, winter and spring), and their interaction, were analyzed with a generalized linear model (GLM, *stats* R package). Additionally, the correlation between the number of NECOs and the number of Franklin’s Gulls (daily maximum number of gulls and NECOs in the CPCV highway) was examined using another GLM (*rstatix* R package [66]), including the year of count as a covariate.

Statistical analyses were performed in R 3.5.1 software [65] with a significance level of α = 0.05.

### Correlations with explanatory variables

Collinearity between five of the six explanatory variables (DS, DSZ, DG, PG and D7) was examined using pairplots (two-way associations) and variance inflation factors (VIF, high-dimensional collinearity, *car* R package [67]). Covariation was assessed using the threshold of 0.65 for correlation coefficient and 3 for VIF. In order to reduce covariation between DG and DS (Pearson correlation, r = 0.8), and between DSZ and DS (r = 0.8), and based on the fact that both DG and DSZ included the most extra information in relation to DS, new variables were created controlling for DS (DG:DS ratio, DSZ:DS ratio). Because the two created variables were highly correlated (r = 0.92), none of them were used in the same model. Each created variable also remained highly correlated in relation to its original variable: DSZ:DS ratio and DSZ (r = 0.95, p < 0.001), DG:DS ratio and DG (r = 0.93, p < 0.001). Finally, D7 showed high correlation in respect to all the other variables excepting PG (r = 0.04), so it was analyzed separately. As a result, three sets of variables were defined: [DS, DG:DS ratio, PG], [DS, DSZ:DS ratio, PG] and [D7, PG]. The variables within each set showed VIFs lower than 2. For each set, different models were tested.

A generalized linear mixed model (GLMM) analysis (*glmmTMB* R package [68]) with a Poisson distribution was conducted for the data of the number of NECOs per pole-cable at 1000 h as the response variable; this count hour was chosen because it registered higher numbers of NECOs during the day and because it was one of the count hours of NECOs’ diurnal activity with a higher occupancy of pole-cables. Different models were generated that included the explanatory variables (see Results), according to the approach of *ad hoc* hypotheses. The code of the pole was included as a random effect to cope with pseudoreplication since each pole-cable was measured repeatedly during the study period. To account for autocorrelation found in the residuals, a first-order autoregressive (AR1) covariance structure was included, with ‘code of the pole + 0’ as the design matrix and the date of count as the grouping factor. For each model, nonsignificant variables (p > 0.05) were excluded and the non-overdispersion assumption was checked. The choice of the most appropriate model was performed through an information-theoretic approach (Akaike information criterion—AIC), taking into account the differences between AIC values (Δ_*I*_, the model with the lowest AIC was considered as the best model when Δ_*I*_ > 2 with respect to the other models) and the Akaike weights (ω_*i*_, [69]).

Unlike the five variables abovementioned, transparency of seawater (T) data were measured by section of the CPCV highway and not by pole-cable. Therefore, the correlation analysis was performed separately, using a logistic regression (logit link function, *stats* R package) with the presence or absence of NECOs on pole-cables as the response variable. For that purpose, on the same day of data collection, the set of 20 pole-cables located perpendicular to each transect was categorized with a value of presence (1) or absence (0) of NECOs.

Correlations and other statistical analyses were performed in R software with a significance level of α = 0.05. The averages are expressed ± 1 SD.

## Results

In the CPCV highway, 651 public lighting poles were identified throughout the six districts. Miraflores and Barranco held 47% of the total poles, since more than half of the total length of the highway corresponds to these districts (6.7 km, Table 1). The largest number of poles were single arm (55%) and double arm (40%) poles, while the rest were no arm poles. In respect of the groynes, Miraflores held 7 of the 12 that exist throughout Costa Verde, followed by Barranco (three groynes) and Chorrillos (two groynes), while in the other districts these rocky structures were absent (Table 1, Fig 1).

**Table 1:** Number and type of lighting poles, length of the highway and number of groynes per district along the Circuito de Playas de la Costa Verde highway in Lima, Peru.

District	Number of poles	Type of pole			Length of the CPCV highway (km)	Number of groynes
		No arm	Single arm	Double arm		
San Miguel	93	0	57	36	2.0	0
Magdalena del Mar	109	0	45	64	2.3	0
San Isidro	57	0	45	12	0.7	0
Miraflores	202	14	97	91	4.6	7
Barranco	105	15	44	46	2.1	3
Chorrillos	85	0	71	14	1.0	2
**TOTAL**	**651**	**29**	**359**	**263**	**12.7**	**12**

### Spatial distribution and temporal variation of the number of NECOs

**Fig 3:**
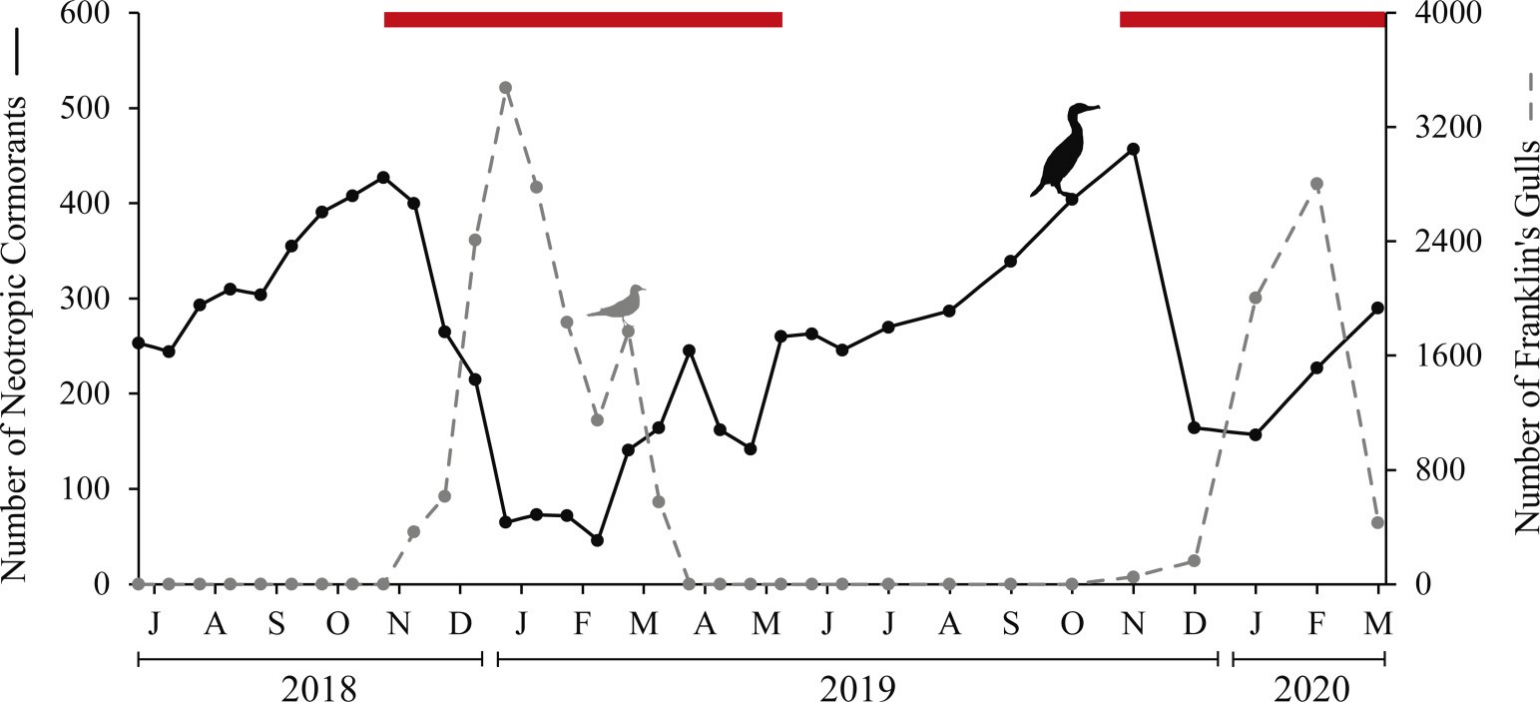
Temporal variation of the daily maximum number of Neotropic Cormorants (*Phalacrocorax brasilianus*) and Franklin’s Gulls (*Leucophaeus pipixcan*) in the Circuito de Playas de la Costa Verde highway in Lima, Peru. The red bars at the top of the graph corresponds to the breeding season of Neotropic Cormorants, which was defined based on records in two coastal wetlands of the central zone of Peru (Los Pantanos de Villa [70] and El Paraíso [71]) and other studies that describe the duration of the stages of their reproductive cycle in different locations of the Peruvian coast [23, 72, 73].

During the entire study period, the average number of NECOs of all the counts in the CPCV highway was 232.4 ± 106.7 individuals. Based on daily maximum numbers of NECOs, the highest number was recorded on November 27, 2019, at 1000 h (457 birds), and the lowest on February 28, 2019, at both 1000 h and 1400 h (46 birds, Fig 3). Of the 651 pole-cables present on this highway, only 17% were occupied by at least one NECO, while the rest were always empty. These occupied pole-cables were located only in the districts of Miraflores (78%) and Barranco (22%).

#### Variations in spatial distribution

The spatial distribution of the NECOs was significantly clustered along the CPCV highway for each of the count hours: 0600 h (global Moran index, GMI = 0.439, z = 11.86, p < 0.001); 1000 h (GMI = 0.416, z = 11.13, p < 0.001); 1400 h (GMI = 0.41, z = 10.97, p < 0.001) and 1800 h (GMI = 0.438, z = 11.83, p < 0.001; Fig 1). At 0600 h and 1800 h, two hotspots were observed, located in Miraflores and Barranco, which gathered 93% of the total number of NECOs (Fig 1). In contrast, at 1000 h and 1400 h, the number of hotspots increased to five (four in Miraflores and one in Barranco) and gathered 84% of the total number of NECOs. The NECOs of the remaining percentage for the four count hours were distributed in other sections of the CPCV highway, on pole-cables with less than two individuals on average (Fig 1). It is important to mention that, despite not having performed surveys during the nighttime, high numbers of NECOs were recurrently observed roosting on pole-cables in the same hotspots as at 1800 h.

**Fig 4:**
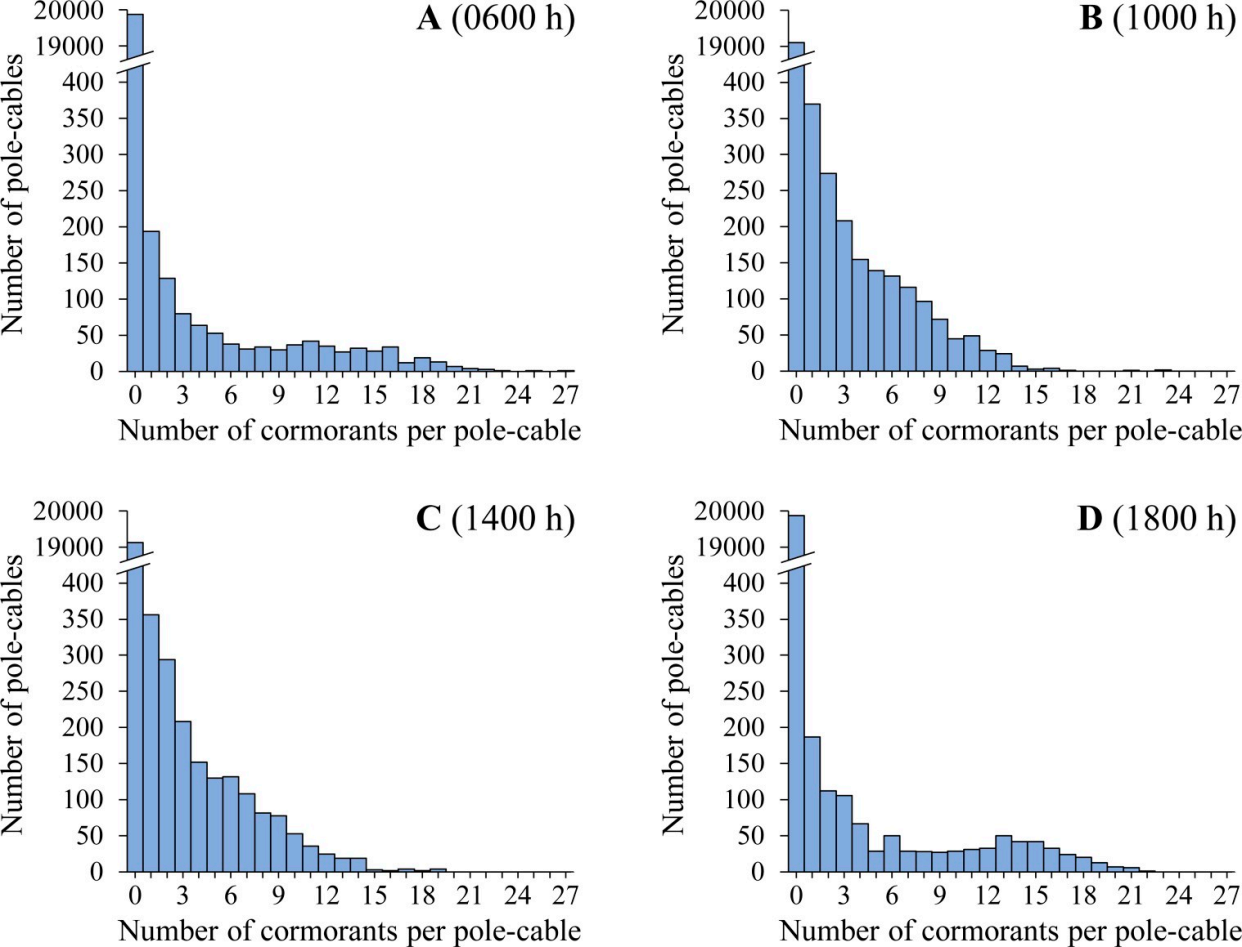
Frequency of the number of Neotropic Cormorants (*Phalacrocorax brasilianus*) per pole-cable, recorded from all surveys during the study period in the Circuito de Playas de la Costa Verde highway in Lima, Peru. (A) 0600 h. (B) 1000 h. (C) 1400 h. (D) 1800 h.

The differences in the number of hotspots reflect changes in the aggregation of NECOs per pole-cable. This aggregation was significantly different only when comparing the count hours with two hotspots (0600 and 1800 h) with respect to the count hours with five hotspots (1000 h and 1400 h; KS test for paired comparisons, [0600 h vs. 1000 h, D = 0.037, p < 0.001], [0600 h vs. 1400 h, D = 0.036, p < 0.001], [1800 h vs. 1000 h, D = 0.037, p < 0.001], [1800 h vs. 1400 h, D = 0.036, p < 0.001]; Fig 4). At 0600 h and 1800 h, there were higher numbers of NECOs per pole-cable; more than 98% of the NECOs recorded in all counts during the study period (cumulative numbers, N_0600 h_ = 6,373 birds, N_1800 h_ = 6,838 birds) occupied pole-cables up to 22 individuals each (Fig 4A and 4D). In contrast, at 1000 h and 1400 h, more than 96% of the NECOs (cumulative numbers, N_1000 h_ = 7,825 birds, N_1400 h_ = 7,728 birds) occupied pole-cables with at most 14 individuals each, so the level of aggregation was lower (Fig 4B and 4C).

#### Temporal variation

**Fig 5:**
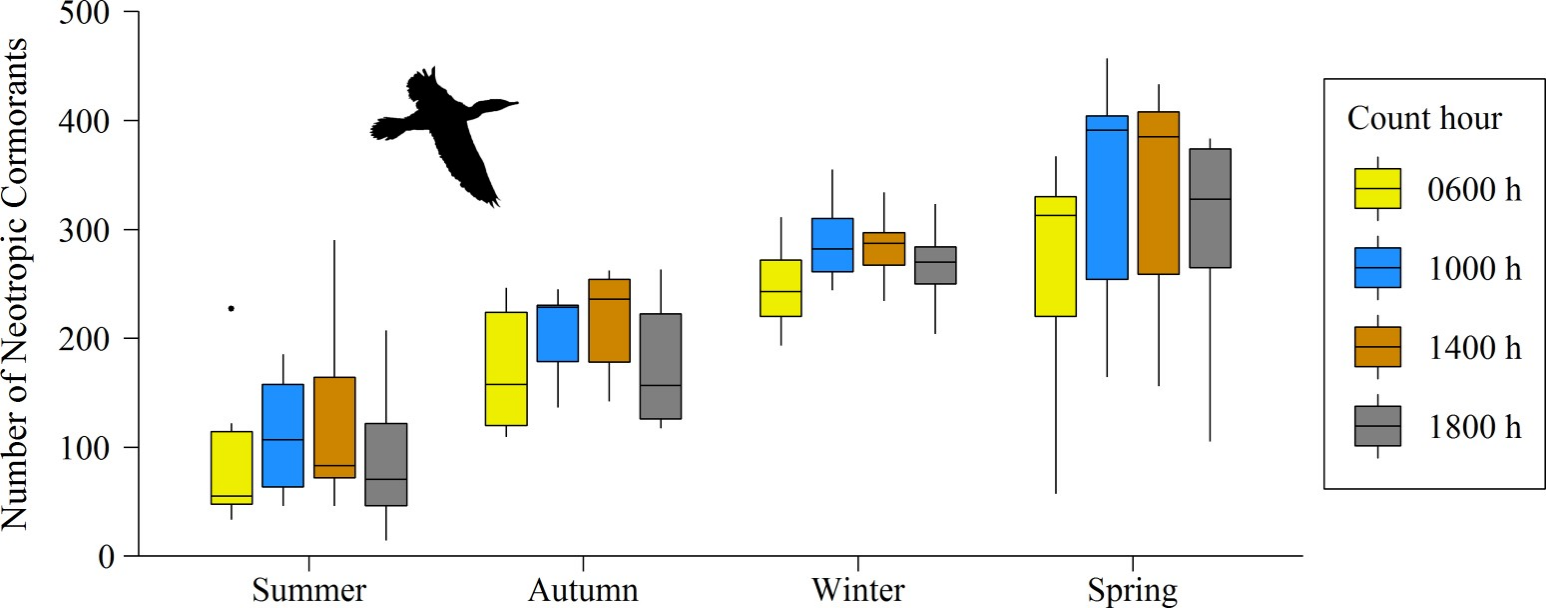
Temporal variation of the number of Neotropic Cormorants (*Phalacrocorax brasilianus*) by count hour and season of the year in the Circuito de Playas de la Costa Verde highway in Lima, Peru.

The number of NECOs in the CPCV highway varied significantly between count hours within the same day (GLM, F_3_ = 3.46, p = 0.019), with the highest numbers of these birds at 1000 h (251.1 ± 110.7 individuals) and at 1400 h (248.4 ± 111.6 individuals, Fig 5). The variation of the number of NECOs between seasons was also significant (GLM, F_3_ = 62.56, p < 0.001; Fig 5). In austral spring (Sep—Nov), a greater number of birds was observed in both 2018 and 2019. In summer (Jan—Feb) of 2019, the number of NECOs was the lowest recorded during the study period (Fig 3). The interaction between count hour and season was not significant (GLM, F_9_ = 0.15, p = 1), showing that the differences in the number of NECOs between count hours were maintained regardless of the season of the year (Fig 5).

Franklin’s Gulls showed a typical pattern of a boreal migratory species. These birds were absent from the CPCV highway in the austral winter of 2018 and in the autumn and winter of 2019 (Fig 3). The first individuals began to appear in late November in both years, reaching peaks of 3,475 and 2,804 individuals during the summer (Jan—Mar) of 2019 and 2020, respectively. There was a significant inverse correlation between the number of NECOs and Franklin’s Gulls (GLM, F_1,30_ = 19.79, p < 0.001), with this relationship being similar between the period 2018–2019 and the period 2019–2020 (GLM, F_1,30_ = 1.98, p = 0.17, Fig 3). It should be noted that during the study period, the perching sites used by Franklin’s Gulls highly matched with those used by NECOs. The presence of Franklin’s Gulls was recorded on 31% of all the pole-cables in the CPCV highway. Moreover, these gulls were observed on 90% of the pole-cables used by the NECOs during the study period.

### Correlations with explanatory variables

**Fig 6:**
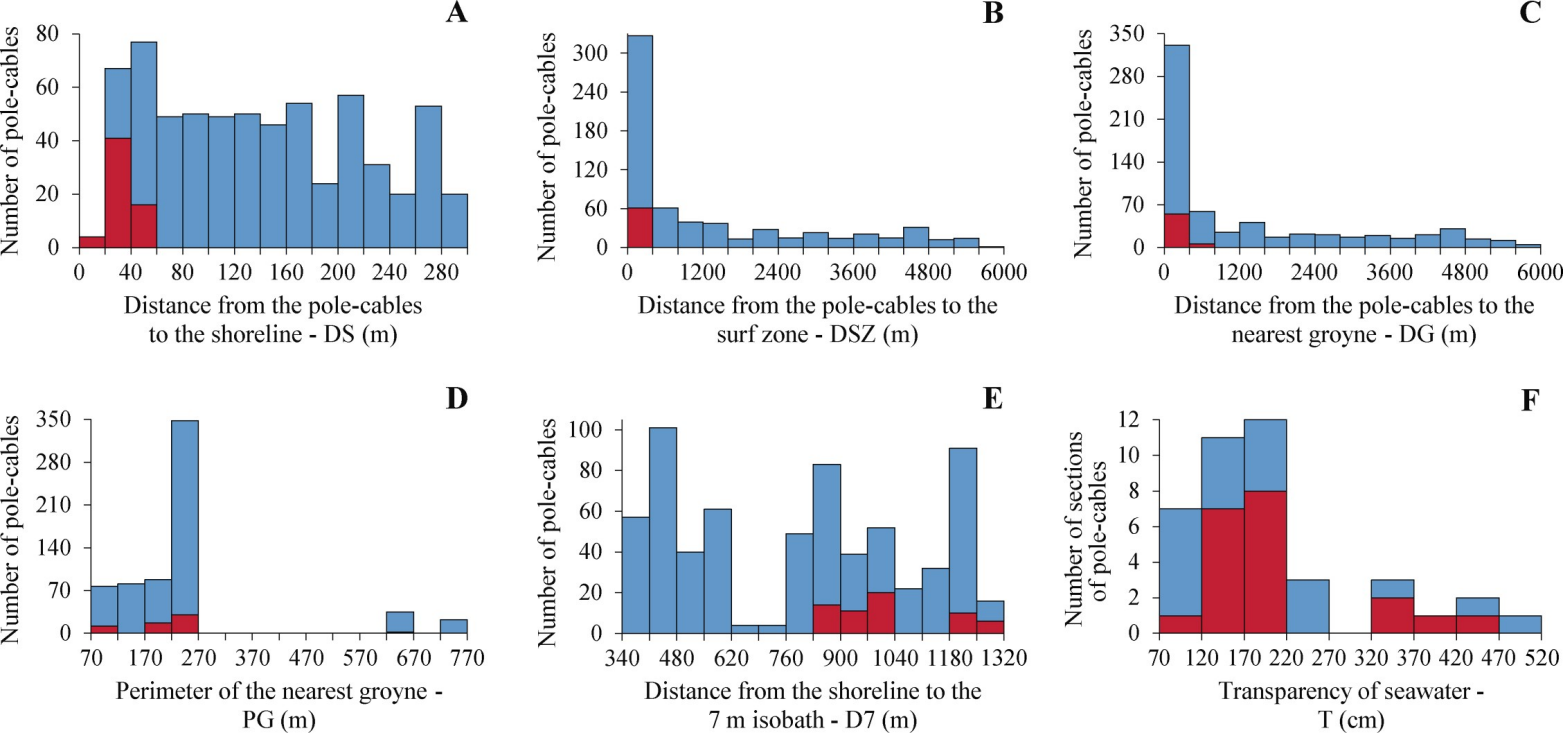
Frequency distributions of the number of pole-cables used (red bars) or not used (blue bars) by Neotropic Cormorants (*Phalacrocorax brasilianus*) at 1000 h in the Circuito de Playas de la Costa Verde highway in Lima, Peru. (A) Distance from the pole-cables to the shoreline. (B) Distance from the pole-cables to the surf zone. (C) Distance from the pole-cables to the nearest groyne. (D) Perimeter of the nearest groyne. (E) Distance from the shoreline to the 7 m isobath. (F) Transparency of seawater.

The distances from the pole-cables to the shoreline (DS) ranged from 16 m to 290 m (Table 2), but the NECOs were located only in pole-cables at distances <60 m (Fig 6A). The distances from the pole-cables to the surf zone (DSZ) presented a distribution very similar to the distances from the pole-cables to the nearest groyne (DG), with 50% of the data included between 18 and 400 m in both cases, and with NECOs perched on pole-cables at distances up to 280 m (DSZ, Fig 6B) and 490 m (DG, Fig 6C). On the other hand, 91% of the pole-cables were located near groynes with a perimeter between 87 and 270 m; the groynes of the remaining percentage (9%) had a perimeter greater than 635 m (Fig 6D). The distance from the shoreline to the 7 m isobath (D7) had a multimodal distribution, with most of the values at approximately 445, 865 and 1215 m; in addition, almost 75% (N = 45) of the pole-cables occupied by NECOs were located in front of distances (D7) between 830 and 995 m, and the rest (N = 16 pole-cables) from 1195 m onwards (Fig 6E). Finally, the transparency of seawater data (T) ranged from 74 cm to 515 cm depth (Table 2) and were distributed asymmetrically to the right, with 75% of the values between 74 and 208 cm depth (Fig 6F); the T values associated with sections of the CPCV highway with presence of NECOs were also skewed to the right (Fig 6F).

**Table 2:** Descriptive statistics of the variables used to explain the spatial distribution of the Neotropic Cormorants (*Phalacrocorax brasilianus*) in the Circuito de Playas de la Costa Verde highway in Lima, Peru.

Variable	N	Mean ± SD	Range	Median
DS (m)	651	138.7 ± 79.9	15.8–289.6	132.3
DSZ (m)	651	1297.9 ± 1600.7	18.5–5615.8	396.5
DG (m)	651	1349.5 ± 1640.3	20.1–5719.3	386.7
PG (m)	12	259.8 ± 201.6	87.4–747.8	196.6
D7 (m)	651	796.6 ± 295.8	349.7–1254.6	843.9
T (cm)	40	200.4 ± 104.4	74.0–514.3	172.5

DS: distance from the pole-cables to the shoreline, DSZ: distance from the pole-cables to the surf zone, DG: distance from the pole-cables to the nearest groyne, PG: perimeter of the groyne, D7: distance from the shoreline to the 7 m isobath, T: transparency of seawater.

According to the Akaike’s information-theoretic approach, the models with individual variables had relatively high AIC values compared to the other models (Table 3), so they could not explain by themselves the spatial distribution of the NECOs in the CPCV highway. In contrast, the best model with lower AIC was M5 (DS [distance to shoreline] + DSZ:DS ratio [distance to surf zone:distance to shoreline ratio] + PG [perimeter of nearest groyne]; Table 3). The other proposed models presented AIC values differences greater than 2 with respect to the M5 model, so they were not taken into account. Additionally, the M5 model was given a higher Akaike weight (ω_*i*_ = 0.67) compared to the other models (Table 3), which reaffirms its choice.

**Table 3:** Poisson generalized linear mixed models to explain the spatial distribution of the Neotropic Cormorants (*Phalacrocorax brasilianus*) in the Circuito de Playas de la Costa Verde highway in Lima, Peru.

Model	Description of the model	AIC	Δ_*i*_	ω_*i*_
M5	DS + DSZ:DS ratio + PG	9861.3	0.0	0.67
M4	DS + DG:DS ratio	9864.2	2.9	0.15
M6	DS + DSZ:DS ratio	9864.3	3.0	0.15
M1	DS	9867.7	6.4	0.03
M2	DSZ:DS ratio	10046.8	185.5	0.0
M3	D7	10048.1	186.8	0.0

AIC: Akaike values, Δ_*i*_: Akaike values differences, ω_*i*_: Akaike weights, DS: distance from the pole-cables to the shoreline, DSZ: distance from the pole-cables to the surf zone, DG: distance from the pole-cables to the nearest groyne, PG: perimeter of the nearest groyne, D7: distance from the shoreline to the 7 m isobath.

According to the chosen model, the number of NECOs per pole-cable was significantly negatively related to DS (Poisson GLMM, β = -0.174 ± 0.017 SE, z = -10.38, p < 0.001), and to DSZ:DS ratio (β = -0.247 ± 0.099 SE, z = -2.497, p = 0.013; Table 4). PG had also a significant but weak negative effect on the number of NECOs per pole-cable compared to the other variables (β = -0.003 ± 0.002 SE, z = -2.229, p = 0.026; Table 4). On the other hand, the correlation between the presence of NECOs on pole-cables and the transparency of seawater (T) was not significant (Logistic regression, *χ*^*2*^Wald = 0.11, p = 0.74).

**Table 4:** Statistical results of the fixed effects of the Poisson model: DS + DSZ:DS ratio + PG, better selected to explain the spatial distribution of the Neotropic Cormorants (*Phalacrocorax brasilianus*) in the Circuito de Playas de la Costa Verde highway in Lima, Peru.

	β estimate	Standard error	z value	p value
**Intercept**	5.965	0.951	6.274	< 0.001
**DS**	-0.174	0.017	-10.38	< 0.001
**DSZ:DS ratio**	-0.247	0.099	-2.497	0.013
**PG**	-0.003	0.002	-2.229	0.026

DS: distance from the pole-cables to the shoreline, DSZ: distance from the pole-cables to the surf zone, PG: perimeter of the nearest groyne.

## Discussion

Our results reveal that the distribution of the NECOs perching on pole-cables along the CPCV highway is neither random nor uniform. In contrast, these birds congregate in a maximum of five hotspots in the districts of Miraflores and Barranco. This study also shows that shorter distances from the perching sites to both the shoreline and the surf zone predict higher numbers of NECOs, which is possibly due to better foraging opportunities for these birds. Our research contributes information that can be used to guide management strategies to mitigate the problems caused by NECOs’ droppings.

### Presence of NECOs in the CPCV highway

In the coast of Peru, NECOs frequently occupy areas with artificial structures where they can perch and/or reproduce, such as metal beams under docks [74], metal ladders that provide access to breakwaters [27], wooden winches (structures to support pulleys) [32, 75], and public lighting poles and telephone cables [29]. They are also found in parks [76] and wetlands [33]. The presence of NECOs in Costa Verde is not a recent event, and their persistence over the years could be explained by the implementation of urban projects in Metropolitan Lima, including its coastal strip [77]. The construction of vehicular roads and touristic/recreational infrastructure has provided these birds with structures for resting, such as the pole-cables along the CPCV highway [29]. In addition, because NECOs do not breed in Costa Verde, their permanency may suggest that this area offers optimal conditions for feeding; the features of the Miraflores Bay may favor the presence of different prey on which these birds feed. It should be noted that possibly part of the population of NECOs has been displaced from nearby natural environments such as Isla El Frontón and Isla San Lorenzo (islands), or from Pantanos de Villa and Humedales de Ventanilla (wetlands), probably due to human disturbance or habitat loss [78, 79], as reported in wetlands of El Salvador [80]. Although the presence of pole-cables in the CPCV highway is advantageous for the NECOs, the costs of using these structures should not be ruled out. During the study period we occasionally observed NECO carcasses on the road, but the mortality rate was not quantified.

### Number of NECOs in the CPCV highway

The daily number of NECOs recorded in the CPCV highway during the study period ranged between 46 and 457 individuals (232.4 ± 106.7 NECOs on average). NECOs are also present on different guano islands and headlands along the coast of Peru, where they roost and breed. Their numbers in 11 of these locations rarely exceed 400 individuals (e.g., [74, 75, 81–83]). For example, between 1990 and 2016, the population of NECOs recorded in Islas Lobos de Afuera and Punta Salinas varied to a maximum of 450 and 472 birds, respectively [81, 84]. These findings are very similar to those found in this study, which suggests that the CPCV highway is an important site for NECOs in the Peruvian coast.

While the daily variation of the number of NECOs in the CPCV highway may respond to diurnal activity patterns related to their foraging behavior, the seasonal variation may be linked to their annual life cycle. The greatest numbers of NECOs on the pole-cables were found between mid-morning and mid-afternoon, suggesting that these birds spend most of the day on these structures resting or in plumage maintenance and drying activities [85–87]. Unlike other regions [42, 85, 86], in the CPCV highway most of the NECOs continued roosting on the pole-cables during the nighttime. It is possible that a low percentage of these birds moved to other overnight places near the CPCV highway (e.g., boat structures in the Chorrillos dock), which could explain their lower number recorded at dawn and dusk. The marked variation of the number of NECOs between seasons in the CPCV highway seems to respond to the phenology of their annual life cycle. Low numbers of birds were recorded from the end of austral spring to the beginning of autumn, which corresponds to their breeding season in the central zone of the Peruvian coast [70, 71]. In addition, adult individuals in breeding plumage were observed in the CPCV highway in November 2018 and December 2019, as well as several juveniles from November 2019 to March 2020 as a result of a possible high reproductive success in 2019. The gradual increase of NECOs from March onwards may be related to a progressive arrival of juveniles and adults from their breeding sites, as happens in other regions [42]. It is important to mention that one of the breeding sites closest to Costa Verde is the wetland Pantanos de Villa [70], located 7–14 km away, so it is feasible that the birds of the CPCV highway move to that area and vice versa. The seasonal variation of the number of NECOs has also been observed in other regions, but with a different phenology [42, 86, 88, 89]. The strong inverse relationship between the number of NECOs and the number of Franklin’s Gulls throughout the study period may be explained by the availability of perching sites left by the NECOs in the CPCV highway during their breeding season (November to May, Fig 3). These boreal migratory gulls arrive at Costa Verde in mid-November, reaching a peak of 2,800–3,480 individuals between December and March, months in which the number of NECOs decreases considerably. Another non-mutually exclusive hypothesis may be a competition between both species for perching space on pole-cables, since there is a high overlap between their perching sites. It should be mentioned that these gulls also occupy pole-cables in other sections of the highway, suggesting that their spatial distribution in the CPCV highway may be explained by factors other than NECOs’.

### Variables related to the distribution patterns of the NECOs in the CPCV highway

This study has shown that higher numbers of NECOs on pole-cables were closely related to a shorter distance from their perching sites to the shoreline. In other regions, the preference of these birds to perch on structures close to coastal and freshwater bodies has also been reported [41, 42, 90]. In the case of the CPCV highway, the 12 m high poles and 8 m high cables, located between 15 and 60 m from the shoreline, would offer the NECOs the best vantage points towards their feeding areas. This could favor the detection of foraging opportunities through the observation of congeners or other predators that also feed at sea [91, 92].

The high and positive linear correlation between DSZ:DS ratio (distance to surf zone:distance to shoreline ratio) and DSZ (distance to surf zone) allowed the use of the latter variable (DSZ) in lieu of the former (DSZ:DS ratio) to interpret the results of the best-supported model. In this sense, closer distances from the pole-cables to the surf zone were associated with higher numbers of NECOs on these perching structures. This behavior may be explained by these areas being particularly biodiverse, and by promoting fish concentration [53, 93]. In fact, during the development of the study, some NECOs were observed swimming and diving among the waves of the surf zone, which agrees with previous observations in Costa Verde [43].

### Management proposals for the problems caused by NECOs’ droppings

Aside from the possible public health problems or corrosion damage to vehicles and infrastructure, we observed and evidenced throughout the study period that the feces of the NECOs do generate nuisance to people, esthetic problems (white spots of excrement on the road and sidewalks) and unpleasant odors, as occurs in coastal cities of Chile [28]. These facts highlight the importance of proposing solutions to this environmental problem.

On the basis of the strategies to limit the interaction between cormorants and fishing activities [94], some of these could be adapted to the case of the NECOs in the CPCV highway. These strategies range from nonlethal deterrent methods to culling. In some regions, the application of the latter has been considered by fish farm managers as an ineffective method against piscivorous birds such as NECOs, because new birds simply replaced individuals that were culled [24, 95]. On the other hand, the use of nonlethal deterrents, such as sound stressors (e.g., sound-emitting bird scarers, and noise-making devices such as cannons, guns and fireworks), has led to the habituation of the NECOs and, therefore, has had a limited effect [28, 95]. It should be mentioned that neither culling nor the use of noise-making projectiles would be adequate for Costa Verde due to the possibility of harming people and because their application would be seen as a controversial practice in terms of both animal protection and public safety. Visual stressors, such as silhouettes that emulate their predators, may also not be effective since no predators of this species are known in Costa Verde. The installation of perch deterrents (e.g., steel spikes, steel inverted-Y structures, dented triangular structures) on the lighting poles could reduce the perching activity of the NECOs, as has been reported with birds of prey on electrical transmission lines [96, 97] and with cormorants and gulls in oyster cages in Canada [98]. Nevertheless, experiences with NECOs on lighting poles on the coastal edge of the city of Antofagasta, Chile, have shown that these birds rest without difficulty on steel spikes and even bend them [28]. Taking into consideration both the preference of the NECOs to settle in areas near the shoreline and the surf zone, and the fact that in Costa Verde the nearest distances between the surf zone and the groynes are relatively short (range from 0 to 154 m), we suggest as a feasible management proposal the construction of structures with multiple perches located on the groynes, so as not to interfere with the activities of people on the beaches. In addition, the droppings in contact with the groynes would be washed out daily by the waves and tides, allowing the fertilization of the sea by increasing primary production with the input of nutrients [99]. Simultaneously, decoys and vocalizations could be used to attract the NECOs to the new structures, which is effective for various seabird species [100, 101]. In the coastal districts of San Bartolo and Punta Hermosa, 40 km south of the CPCV highway, NECOs perch on lighting poles on groynes, despite the availability of other poles along boulevards close to the shore. This evidences that a relocation of the NECOs to perching structures on groynes may work.

## Conclusions

This study is the first to elucidate the factors that influence the spatial distribution of a seabird species in a coastal urban area in Peru. The presence of NECOs on pole-cables is not unnoticed in Costa Verde since their droppings come into contact with areas of high levels of human activity. Despite this fact, municipal action has not yet been taken. However, with the development of new urban projects in Costa Verde (e.g., on February 27, 2020, an additional northern section of 2.7 km of the CPCV highway was inaugurated), the information provided by this research becomes increasingly relevant for better planning and reduction of conflicts between NECOs and people. The most feasible management proposal that we suggest based on the results of this study is to discourage these birds from using the poles and cables of the CPCV highway and at the same time relocate them to new perching structures on nearby groynes. In order to test the effectiveness of this proposal in NECOs relocation, pilot projects must be conducted in a small portion of the CPCV highway containing groynes and a hotspot of NECOs perching on pole-cables. Additionally, continuation of monthly monitoring of the number of NECOs in the CPCV highway, and characterization of the microbiota in their excrement are recommended. It is important to mention that NECOs are also present in other urban areas and ports along the Peruvian coast, generating conflicts similar to those shown in this study. Thus, the approach used in the CPCV highway can provide guidance for other NECO-human conflict areas along the Peruvian coast.

## References

[1] Dias MP, Martin R, Pearmain EJ, Burfield IJ, Small C, Phillips RA, et al. Threats to seabirds: A global assessment. Conserv Biol. 2019;237: 525–537. 10.1016/j.biocon.2019.06.033

[2] Croxall JP, Butchart SHM, Lascelles B, Stattersfield AJ, Sullivan B, Symes A, et al. Seabird conservation status, threats and priority actions: A global assessment. Bird Conserv Int. 2012;22(1): 1–34. 10.1017/s0959270912000020

[3] BirdLife International. State of the world’s birds: Taking the pulse of the planet. Cambridge: BirdLife International; 2018.

[4] Pedrocchi V, Oro D, González-Solís J, Ruiz X, Jover Ll. Differences in diet between the two largest breeding colonies of Audouin’s Gulls: The effects of fishery activities. Sci Mar. 2002;66(3): 313–320.

[5] González D, Marinao C, Yorio P. Importancia de los descartes pesqueros en la dieta de la gaviota cocinera (*Larus dominicanus*) en el Golfo San Jorge, Patagonia. Ornitol Neotrop. 2017;28: 103–111.

[6] Sherley RB, Ladd‐Jones H, Garthe S, Stevenson O, Votier SC. Scavenger communities and fisheries waste: North Sea discards support 3 million seabirds, 2 million fewer than in 1990. Fish Fish. 2019;00: 1–14. 10.1111/faf.12422

[7] Giaccardi M, Yorio P, Lizurume ME. Patrones estacionales de abundancia de la gaviota cocinera (*Larus dominicanus*) en un basural patagónico y sus relaciones con el manejo de residuos urbanos y pesqueros. Ornitol Neotrop. 1997;8: 77–84.

[8] Fuirst M, Veit RR, Hahn M, Dheilly N, Thorne LH. Effects of urbanization on the foraging ecology and microbiota of the generalist seabird *Larus argentatus*. PLoS ONE. 2018;13(12): e0209200. 10.1371/journal.pone.0209200 30562368 PMC6298667

[9] Roby DD, Collis K, Lyons DE, Craig DP, Adkins JY, Myers AM, et al. Effects of colony relocation on diet and productivity of Caspian Terns. J Wildl Manage. 2002;66(3): 662–673. 10.2307/3803132

[10] Weitzman J, Steeves L, Bradford J, Filgueira R. Far-field and near-field effects of marine aquaculture. In: Sheppard C, editor. World seas: An environmental evaluation. 2nd ed. Vol. III: Ecological issues and environmental impacts. Academic Press; 2019. pp. 197–220. 10.1016/B978-0-12-805052-1.00011–5

[11] Furness RW, Monaghan P. Seabird ecology. 1st ed. Glasgow and London: Blackie; 1987.

[12] Contreras AJ, Tejeda AG, García JA. Las aves como plaga, controles y manejo. Ciencia UANL. 2003;6(1): 93–98.

[13] Diel DG, Miller PJ, Wolf PC, Mickley RM, Musante AR, Emanueli DC, et al. Characterization of Newcastle disease viruses isolated from cormorant and gull species in the United States in 2010. Avian Dis. 2012;56(1): 128–133. 10.1637/9886-081111-Reg.1 22545538

[14] Mukerji S, Stegger M, Truswell AV, Laird T, Jordan D, Abraham RJ, et al. Resistance to critically important antimicrobials in Australian Silver Gulls (*Chroicocephalus novaehollandiae*) and evidence of anthropogenic origins. J Antimicrob Chemother. 2019;74: 2566–2574. 10.1093/jac/dkz242 31287537

[15] Franklin AB, Ramey AM, Bentler KT, Barrett NL, McCurdy LM, Ahlstrom CA, et al. Gulls as sources of environmental contamination by colistin-resistant bacteria. Sci Rep. 2020;10: 4408. 10.1038/s41598-020-61318-2 32157139 PMC7064522

[16] Mallory ML, Mahon L, Tomlik MD, White C, Milton GR, Spooner I. Colonial Marine birds influence island soil chemistry through biotransport of trace elements. Water Air Soil Pollut. 2015;226: 31. 10.1007/s11270-015-2314-9

[17] Belant JL. Gulls in urban environments: Landscape-level management to reduce conflict. Landscape Urban Plan. 1997;38: 245–258. 10.1016/s0169-2046(97)00037-6

[18] Rock P. Urban gulls: Problems and solutions. Br Birds. 2005;98: 338–355.

[19] Sodhi NS. Competition in the air: Birds versus aircraft. Auk. 2002;119(3): 587–595. 10.2307/4089960

[20] Kitowski I. Civil and military birdstrikes in Europe: An ornithological approach. J Appl Sci. 2011;11(1): 183–191. 10.3923/jas.2011.183.191

[21] Silva MP, Bastida R, Darrieu C. Dieta de la gaviota cocinera (*Larus dominicanus*) en zonas costeras de la provincia de Buenos Aires, Argentina. Ornitol Neotrop. 2000;11: 331–339.

[22] Orta J. Family Phalacrocoracidae (cormorants). In: Del Hoyo J, Elliott A, Sargatal J, editors. Handbook of the birds of the world. Vol. 1: Ostrich to ducks. Barcelona: Lynx Edicions; 1992. pp. 326–353.

[23] Johnsgard PA. Cormorants, darters and pelicans of the world. 1st ed. Washington DC: Smithsonian Institution Press; 1993.

[24] Guevara EA, Santander T, Mueces T, Terán K, Henry P. Population growth and seasonal abundance of the Neotropic Cormorant (*Phalacrocorax brasilianus*) at highland lakes in Ecuador. Waterbirds. 2011;34(4): 499–503. 10.1675/063.034.0413

[25] Burr PC, Avery JL, Street GM, Strickland BK, Dorr BS. Piscivorous bird use of aquaculture and natural water bodies in Mississippi. J Wildl Manage. 2020; 1–10. 10.1002/jwmg.21948

[26] Marzano M, Carss DN, Cheyne I. Managing European cormorant-fisheries conflicts: Problems, practicalities and policy. Fish Manag Ecol. 2013;20(5): 401–413. 10.1111/fme.12025

[27] Chamorro A, Contreras C, Raschio G. Evaluación rápida del ecosistema marino desarrollado en la zona de carga de la Planta Pampa Melchorita de Perú LNG. Report. Lima: Ecosystem Services LLC; 2014 Jul. Available from: http://hdl.handle.net/1834/8420

[28] Guerra-Correa C, Guerra-Castro Ch, Páez-Godoy J. Diagnóstico, plan de seguimiento y control de cormorán yeco (*Phalacrocorax brasilianus*) en la ciudad de Antofagasta. Report. Chile: Centro Regional de Estudios y Educación Ambiental, Universidad de Antofagasta; 2013. Available from: http://yecoproyecto.wix.com/yeco#!documentos

[29] Majluf P (Fundación Cayetano Heredia, Lima). Identificación de ecosistemas y servicios ecosistémicos dentro del ámbito de la Costa Verde. Report. Lima: Centro para la Sostenibilidad Ambiental de la Universidad Peruana Cayetano Heredia; 2014 Jul. Proceso de Selección N° 002-2014-MML-APCV-GA/AA. Available from: http://hdl.handle.net/1834/8419

[30] Murphy RC. Oceanic birds of South America: A study of species of the related coasts and seas, including the American quadrant of Antarctica, based upon the Brewster-Sanford collection in the American Museum of Natural History. Vol. II. New York: The Macmillan Company, The American Museum of Natural History; 1936. 10.5962/bhl.title.11916

[31] Tovar H. Áreas de reproducción y distribución de las aves marinas en el litoral peruano. Bol Inst Mar Perú. 1968;1(10): 524–546.

[32] Tovar H, Cabrera D. Conservación y manejo de aves guaneras. Lima: Asamblea Nacional de Rectores, Universidad Nacional Agraria La Molina; 2005.

[33] Barrio J, Guillén C. Aves de los humedales de la costa peruana. 1st ed. Lima: Corbidi; 2014.

[34] Goodall JD, Johnson AW, Philippi RA. Las aves de Chile, su conocimiento y sus costumbres. Buenos Aires: Platt Establecimientos Gráficos; 1951.

[35] Coker RE. Habits and economic relations of the guano birds of Peru. Proc US Nat Mus. 1919;56(2298): 449–529. 10.5479/si.00963801.56–2298.449

[36] Quintana F, Yorio P, Lisnizer N, Gatto A, Soria G. Diving behavior and foraging areas of the Neotropic Cormorant at a marine colony in Patagonia, Argentina. Wilson Bull. 2004;116(1): 83–88. 10.1676/0043-5643(2004)116[0083:DBAFAO]2.0.CO;2

[37] Fagan J, Komar O. Peterson field guide to birds of Northern Central America: Belize, El Salvador, Guatemala, Honduras. New York: Houghton Mifflin Harcourt; 2016.

[38] Muñoz J, Marín G, Andrade J, Alzola R. Notas sobre la dieta de la cotúa olivácea (*Phalacrocorax olivaceus*) en una laguna marino-costera de la Península de Araya, Venezuela. Saber. 2008;20(2): 253–258.

[39] Petracci PF, Cereghetti J, Martín J, Obed YS. Dieta del biguá (*Phalacrocorax olivaceus*) durante la primavera en el estuario de Bahía Blanca, Buenos Aires, Argentina. Hornero. 2009;24(2): 73–78.

[40] Muñoz-Gil J, Marín-Espinoza G, Andrade-Vigo J, Zavala R, Mata A. Trophic position of the Neotropic Cormorant (*Phalacrocorax brasilianus*): Integrating diet and stable isotope analysis. J Ornithol. 2012;154: 13–18. 10.1007/s10336-012-0863-x

[41] Morrison ML, Douglas R, Shanley E Jr. Age and foraging ability relationships of Olivaceous Cormorants. Wilson Bull. 1978;90(3): 414–422. 10.2307/4161091

[42] Barquete V, Vooren CM, Bugoni L. Seasonal abundance of the Neotropic Cormorant (*Phalacrocorax brasilianus*) at Lagoa dos Patos estuary, Southern Brazil. Hornero. 2008;23(1): 15–22.

[43] Koepcke M. Corte ecológico transversal en los Andes del Perú central con especial consideración de las aves. Parte I: Costa, Vertientes occidentales y Región altoandina. Lima: Universidad Nacional Mayor de San Marcos; 1954.

[44] Castillo R (Convenio Autoridad del Proyecto Costa Verde (APCV)—Instituto Metropolitano de Planificación (IMP), Lima). Plan maestro de desarrollo de la Costa Verde 1995–2010. Vol. A, D. Report. Lima: Municipalidad Metropolitana de Lima; 1995 Aug. Acuerdo de Consejo No. 079 APCV. Available from: http://www.apcvperu.gob.pe/index.php/plan-maestro

[45] Verdugo C, Pinto A, Ariyama N, Moroni M, Hernandez C. Molecular identification of avian viruses in Neotropic Cormorants (*Phalacrocorax brasilianus*) in Chile. J Wildl Dis. 2019;55(1): 105–112. 10.7589/2017-10-256 30216128

[46] Contreras A, Gómez-Martín A, Paterna A, Tatay-Dualde J, Prats-Van Der Ham M, Corrales JC, et al. Papel epidemiológico de las aves en la transmisión y mantenimiento de zoonosis. Rev Sci Tech. 2016;35(3): 845–862. 10.20506/rst.35.3.2574 28332645

[47] Banse T. Cormorants are protected. But their poop is corroding a bridge across the Columbia River. KUOW Public Radio. 2019 6 12 [Cited 2020 Feb 12]. Available from: https://www.kuow.org/stories/cormorants-flock-by-the-thousands-to-a-columbia-river-bridge-where-they-re-unwanted

[48] Frankowicz K. On the Astoria Bridge, a cormorant kingdom grows. Thousands of birds at home on the span. The Astorian. 2019 5 31 [Cited 2019 Nov 1]. Available from: https://www.dailyastorian.com/news/local/on-the-astoria-bridge-acormorant-kingdom-grows/article_97d11218-823b-11e9-99f7-af1fcb15e4ee.html

[49] Casaux RJ, Di Prinzio CY, Bertolin ML, Tartara MA. Diet of the Neotropic Cormorant *Phalacrocorax olivaceus* at West Chubut, Patagonia, Argentina. Waterbirds. 2009;32(3): 444–449. 10.1675/063.032.0310

[50] Esri Inc., Garmin International Inc., U.S. Central Intelligence Agency. World Countries [shapefile]. c2017 [updated 2020 Feb 11; cited 2020 May 27]. In: Esri Data & Maps [Internet]. Available from: https://www.arcgis.com/home/item.html?id = d974d9c6bc924ae0a2ffea0a46d71e3d

[51] Esri Inc. ArcGIS Desktop. Version 10.5 [software]. Redlands, California: Environmental Systems Research Institute; 2016. Available from: https://desktop.arcgis.com/es/arcmap/

[52] Dally WR. Surf zone processes. In: Finkl CW, Makowski C, editors. Encyclopedia of coastal science. Living ed. Encyclopedia of earth sciences series. Cham: Springer; 2018. 10.1007/978-3-319-48657-4_306–2

[53] McLachlan A, Defeo O. The ecology of sandy shores. 3rd ed. London: Academic Press; 2018. 10.1016/B978-0-12-809467-9.00010–2

[54] Google LLC. Google Earth Pro. Version 7.3 [software]. Mountain View, California: Google LLC; 2019. Available from: https://www.google.es/earth/versions/

[55] Martin D, Bertasi F, Colangelo MA, de Vries M, Frost M, Hawkins SJ, et al. Ecological impact of coastal defence structures on sediment and mobile fauna: Evaluating and forecasting consequences of unavoidable modifications of native habitats. Coast Eng. 2005;52(10–11): 1027–1051. 10.1016/j.coastaleng.2005.09.006

[56] Dugan JE, Airoldi L, Chapman MG, Walker SJ, Schlacher T. Estuarine and coastal structures: Environmental effects, a focus on shore and nearshore structures. In: Wolanski E, McLusky D, editors. Treatise on estuarine and coastal science. Vol. 8: Human-induced problems (uses and abuses). London: Academic Press; 2011. pp. 17–41. 10.1016/B978-0-12-374711-2.00802–0

[57] Anton IA, Panaitescu M, Panaitescu F-V, Ghiţă S. Impact of coastal protection systems on marine ecosystems. E3S Web Conf. 2019;85: 07011. 10.1051/e3sconf/20198507011

[58] Morrison ML, Shanley E Jr, Slack RD. The food of nestling Olivaceous Cormorants. Southwest Nat. 1977;22(3): 321–326. 10.2307/30054799

[59] Marina de Guerra del Perú, Dirección de Hidrografía y Navegación. Bahía Miraflores [nautical chart]. Callao: Marina de Guerra del Perú; 2013. Available from: https://www.dhn.mil.pe/vemana/index.php?cat = Productos

[60] Duffy DC, Wilson RP, Wilson M-P, Velasquez C. Plunge-diving by Olivaceous Cormorants in Chile. Wilson Bull. 1986;98(4): 607–608.

[61] Gatto A, Quintana F, Yorio P. Feeding behavior and habitat use in a waterbird assemblage at a marine wetland in Coastal Patagonia, Argentina. Waterbirds. 2008;31(3): 463–471. 10.1675/1524-4695-31.3.463

[62] Shealer DA. Foraging behavior and food of seabirds. In: Schreiber EA, Burger J, editors. Biology of marine birds. 1st ed. Boca Raton (FL): CRC Press; 2001. pp. 137–177. 10.1201/9781420036305

[63] Henkel LA. Effect of water clarity on the distribution of marine birds in nearshore waters of Monterey Bay, California. J Field Ornithol. 2006;77(2): 151–156. 10.1111/j.1557-9263.2006.00035.x

[64] Instituto Geográfico Nacional. Límites Distritales [shapefile]. c2019 [cited 2019 Mar 19]. In: Infraestructura Nacional de Datos Geoespaciales Fundamentales del Perú [Internet]. Available from: http://www.idep.gob.pe/#visor

[65] R Core Team. R: A language and environment for statistical computing. Version 3.5.1 [software]. Vienna, Austria: The R Foundation for Statistical Computing; 2018. Available from: https://www.R-project.org/

[66] Kassambara A. rstatix: Pipe-friendly framework for basic statistical tests. Version 0.4.0 [R package]. 2020. Available from: https://CRAN.R-project.org/package=rstatix

[67] Fox J, Weisberg S. An {R} companion to applied regression. 3rd ed. Thousand Oaks (CA): Sage; 2019.

[68] Brooks ME, Kristensen K, van Benthem KJ, Magnusson A, Berg CW, Nielsen A, et al. glmmTMB balances speed and flexibility among packages for zero-inflated generalized linear mixed modeling. R J. 2017;9(2): 378–400. 10.3929/ETHZ-B-000240890

[69] Burnham KP, Anderson DR. Model selection and multimodel inference: A practical information-theoretic approach. 2nd ed. New York: Springer; 2002. 10.1007/b97636

[70] Amaro L, Goyoneche G. Anidación de aves en el Refugio de Vida Silvestre Los Pantanos de Villa 2007–2009, Lima-Perú. The Biologist (Lima). 2017;15(1): 155–171. 10.24039/rtb2017151151

[71] Quiñonez AS, Hernandez F. Uso de hábitat y estado de conservación de las aves en el humedal El Paraíso, Lima, Perú. Rev Peru Biol. 2017;24(2): 175–186. 10.15381/rpb.v24i2.13494

[72] Galarza N. Informe sobre estudios ornitológicos realizados en el laboratorio de La Puntilla (Pisco) en setiembre de 1965/66. Report. Lima: Instituto del Mar del Perú; 1968 Sep. Serie de Informes Especiales N° IM-31. Available from: http://biblioimarpe.imarpe.gob.pe/handle/123456789/1461

[73] Stucchi M, Figueroa J, Mori G, Flores F. Revisión y actualización de la avifauna de las islas Lobos de Afuera (Perú). Bol Inf UNOP. 2011;6(1): 14–27.

[74] Figueroa J, Roca M, Torres D, Guillermo E, Paredes F, Barraza D. Caracterización de la fauna terrestre: Aves, mamíferos y reptiles. In: Servicio Nacional de Áreas Naturales Protegidas por el Estado (SERNANP), editor. Línea base biológica terrestre y marina de la Reserva Nacional Sistema de Islas, Islotes y Puntas Guaneras—Punta Coles (Moquegua). 1st ed. Lima: SERNANP; 2016. pp. 13–114.

[75] Figueroa J, Guillermo E, Álvarez A, Ipanaqué M, Hernández W, Valdivia L. Caracterización de la fauna terrestre de la isla Don Martín: Aves, mamíferos y reptiles. In: Servicio Nacional de Áreas Naturales Protegidas por el Estado (SERNANP), editor. Línea base biológica terrestre y marina de la Reserva Nacional Sistema de Islas, Islotes y Puntas Guaneras—Islote Don Martín (Lima). 1st ed. Lima: SERNANP; 2019. pp. 9–111.

[76] Carazas N, Salazar R, Podestá J. Avifauna silvestre del Parque de las Leyendas, Lima, Perú. The Biologist (Lima). 2019;17(1): 19–30. 10.24039/rtb2019171289

[77] Castillo RF. La planificación urbana de Lima-Callao 1949–2013: Del urbanismo funcionalista a la planificación del desarrollo urbano sostenible. Paideia XXI. 2013;3(4): 20–32. 10.31381/paideia.v3i4.925

[78] Aponte H, Ramírez W. Riqueza florística y estado de conservación del Área de Conservación Regional Humedales de Ventanilla, Callao, Perú. The Biologist (Lima). 2014;12(2): 270–282. 10.24039/rtb2014122356

[79] Pulido VM, Bermúdez L. Estado actual de la conservación de los hábitats de los Pantanos de Villa, Lima, Perú. Arnaldoa. 2018;25(2): 679–702. 10.22497/arnaldoa.252.25219

[80] Herrera N, Ibarra R, Salinas M. Distribución, abundancia y anidación del cormorán neotropical (*Phalacrocorax brasilianus*) en El Salvador. Mesoamericana. 2008;12(1): 24–31.

[81] Figueroa J, Llica M, Ordoñez J, Chugnas L, Salinas V, Flores H, et al. Caracterización de la fauna terrestre de Punta Salinas, Isla Huampanú e Isla Mazorca: Aves, mamíferos y reptiles. In: Servicio Nacional de Áreas Naturales Protegidas por el Estado (SERNANP), editor. Línea base biológica terrestre y marina de la Reserva Nacional Sistema de Islas, Islotes y Puntas Guaneras—Punta Salinas, Isla Huampanú e Isla Mazorca (Lima). 1st ed. Lima: SERNANP; 2019. pp. 10–208.

[82] Figueroa J, Tomairo M, Flores A, Jaime M, Melgarejo I, Hernández W, et al. Caracterización de la fauna terrestre de las Islas Guañape: Aves, mamíferos y reptiles. In: Servicio Nacional de Áreas Naturales Protegidas por el Estado (SERNANP), editor. Línea base biológica terrestre y marina de la Reserva Nacional Sistema de Islas, Islotes y Puntas Guaneras—Islas Guañape (La Libertad). 1st ed. Lima: SERNANP; 2019. pp. 10–137.

[83] Aguilar RE, Llapapasca MA, Quiñones J, Rivadeneyra SB, Torres D. Depredadores superiores en Isla Lobos de Tierra, Perú. Evaluación de línea base (GEF-UNDP 2014). Inf Inst Mar Perú. 2020;47(1): 37–64.

[84] Figueroa J, Timaná L, Gutiérrez C, Roca M, Hernández W, Ramírez R. Caracterización de la fauna terrestre de las Islas Lobos de Afuera: Aves, mamíferos y reptiles. In: Servicio Nacional de Áreas Naturales Protegidas por el Estado (SERNANP), editor. Línea base biológica terrestre y marina de la Reserva Nacional Sistema de Islas, Islotes y Puntas Guaneras—Islas Lobos de Afuera (Lambayeque). 1st ed. Lima: SERNANP; 2019. pp. 8–132.

[85] Branco JO, Braun JRR, Verani JR. Seasonal variation in the abundance of seabirds in areas of mariculture. Braz Arch Biol Technol. 2001;44(4): 395–399. 10.1590/s1516-89132001000400009

[86] Branco JO, Evangelista CL, Lunardon-Branco MJ, Azevedo-Júnior SM, Larrazábal ME. Atividade diária de *Phalacrocorax brasilianus* (Aves, Phalacrocoracidae), na região do Saco da Fazenda, Itajaí, SC, Brasil. Ornithologia. 2009;3(2): 73–82.

[87] Da Silva TL, Cabral RBG, Ferreira I. Behavior and seasonal abundance of Neotropic Cormorant *Nannopterum brasilianus* (Gmelin, 1789) in southeastern, Brazil. Rev Bras Ornitol. 2018;26(4): 219–226. 10.1007/BF03544434

[88] Branco JO. Flutuações sazonais na abundância de *Phalacrocorax brasilianus* (Gmelin) no estuário do Saco da Fazenda, Itajaí, Santa Catarina, Brasil. Rev Bras Zool. 2002;19(4): 1057–1062. 10.1590/S0101-81752002000400010

[89] Barbieri E, Paes ET. The birds at Ilha Comprida beach (São Paulo state, Brazil): A multivariate approach. Biota Neotrop. 2008;8(3): 41–50. 10.1590/S1676-06032008000300003

[90] Rutigliano A. Aves y mamíferos del cantón Cotacachi. 1st ed. Cotacachi: Asamblea de Unidad Cantonal de Cotacachi (AUC); 2006.

[91] Weimerskirch H, Bertrand S, Silva J, Marques JC, Goya E. Use of social information in seabirds: Compass rafts indicate the heading of food patches. PLoS ONE. 2010;5(3): e9928. 10.1371/journal.pone.0009928 20360959 PMC2847911

[92] Thiebault A, Mullers RHE, Pistorius PA, Tremblay Y. Local enhancement in a seabird: Reaction distances and foraging consequence of predator aggregations. Behav Ecol. 2014;25(6): 1302–1310. 10.1093/beheco/aru132

[93] Olds AD, Vargas-Fonseca E, Connolly RM, Gilby BL, Huijbers CM, Hyndes GA, et al. The ecology of fish in the surf zones of ocean beaches: A global review. Fish Fish. 2017;19(1): 78–89. 10.1111/faf.12237

[94] Russell I, Broughton B, Keller T, Carss D. The INTERCAFE cormorant management toolbox: Methods for reducing Cormorant problems at European fisheries. INTERCAFE COST Action 635 Final Report III. NERC Centre for Ecology & Hydrology on behalf of COST; 2012. 10.13140/2.1.5061.2481

[95] Bechard MJ, Márquez-Reyes C. Mortality of wintering Ospreys and other birds at aquaculture facilities in Colombia. J Raptor Res. 2003;37(4): 292–298.

[96] Lammers WM, Collopy MW. Effectiveness of avian predator perch deterrents on electric transmission lines. J Wildl Manage. 2007;71(8): 2752–2758. 10.2193/2005-752

[97] Slater SJ, Smith JP. Effectiveness of raptor perch deterrents on an electrical transmission line in southwestern Wyoming. J Wildl Manage. 2010;74(5): 1080–1088. 10.2193/2008-525

[98] Comeau LA, St‐Onge P, Pernet F, Lanteigne L. Deterring coastal birds from roosting on oyster culture gear in eastern New Brunswick, Canada. Aquac Eng. 2009;40: 87–94. 10.1016/j.aquaeng.2008.11.003

[99] Gil-Weir K, Weir E, Casler CL, Aniyar S. Ecological functions and economic value of the Neotropic Cormorant (*Phalacrocorax brasilianus*) in Los Olivitos Estuary, Venezuela. Environ Dev Econ. 2011;16(5): 553–572. 10.1017/S1355770X11000179

[100] Kress SW. The use of decoys, sound recordings, and gull control for re-establishing a tern colony in Maine. Col Waterbirds. 1983;6: 185–196. 10.2307/1520987

[101] Williams DR, Pople RG, Showler DA, Dicks LV, Child MF, zu Ermgassen EKHJ, et al. Bird conservation: Global evidence for the effects of interventions. Synopses of conservation evidence series. Exeter: Pelagic Publishing; 2012. 10.13140/2.1.1927.3924

